# STransU2Net: Transformer based hybrid model for building segmentation in detailed satellite imagery

**DOI:** 10.1371/journal.pone.0299732

**Published:** 2024-09-12

**Authors:** Guangjie Liu, Kuo Diao, Jinlong Zhu, Qi Wang, Meng Li

**Affiliations:** 1 College of Computer Science and Technology, Changchun Normal University, Changchun, Jiling, China; 2 College of Data Intelligence, Yantai Institute of Science and Technology, Yantai, Shandong, China; 3 China grain reserves group ltd.company, Changchun, Jiling, China; Taipei Medical University, TAIWAN

## Abstract

As essential components of human society, buildings serve a multitude of functions and significance. Convolutional Neural Network (CNN) has made remarkable progress in the task of building extraction from detailed satellite imagery, owing to the potent capability to capture local information. However, CNN performs suboptimal in extracting larger buildings. Conversely, Transformer has excelled in capturing global information through self-attention mechanisms but are less effective in capturing local information compared to CNN, resulting in suboptimal performance in extracting smaller buildings. Therefore, we have designed the hybrid model STransU2Net, which combines meticulously designed Transformer and CNN to extract buildings of various sizes. In detail, we designed a Bottleneck Pooling Block (BPB) to replace the conventional Max Pooling layer during the downsampling phase, aiming to enhance the extraction of edge information. Furthermore, we devised the Channel And Spatial Attention Block (CSAB) to enhance the target location information during the encoding and decoding stages. Additionally, we added a Swin Transformer Block (STB) at the skip connection location to enhance the model’s global modeling ability. Finally, we empirically assessed the performance of STransU2Net on both the Aerial imagery and Satellite II datasets, The IoU achieved state-of-the-art results with 91.04% and 59.09%, respectively, outperforming other models.

## Introduction

Building information constitutes a crucial element within detailed satellite imagery and assumes a pivotal function across diverse domains, such as urban planning [1–3], economic activity assessment [4, 5], and disaster information statistics [6, 7]. However, the challenges inherent in building segmentation stem from significant disparities in building characteristics, encompassing factors such as size, form, hue, texture, and other factors. Consequently, accurately extracting building information extraction from detailed satellite imagery has garnered significant attention within the academic community [8, 9].

Traditional methods for building segmentation in detailed satellite imagery depend on low-level characteristics, such as edge, gray level, texture, and so on [10–13]. For example, the threshold segmentation method based on image pixels [14], although efficient and computationally inexpensive, is sensitive to noise and performs poorly in extracting complex buildings. Similarly, machine learning-based segmentation methods [15–18] such as clustering segmentation [19] do not require training, but their segmentation results are overly dependent on initial parameters and perform poorly when dealing with complex detailed satellite imagery.

In the past few years, propelled by the continual advancements in deep learning and computing hardware, deep learning algorithms leveraging CNN has comprehensively surpassed conventional methods. These algorithms have made remarkable strides in various computer vision domains, including image classification, object detection, and image segmentation, among others. In 2015, Long et al. introduced the fully convolutional network (FCN) [20], which marked a significant milestone in utilizing convolutional neural networks for image semantic segmentation. FCN substitutes fully connected layers with convolutional layers and utilizes feature maps generated by intermediate pooling layers. These feature maps are then upsampled using linear interpolation to match the original input size, enabling arbitrary-sized image input and achieving end-to-end pixel-level prediction, but the design of its decoding phase is overly simplistic, subsequently proposed networks have made significant improvements in this regard.

Since the introduction of FCN, researchers have conducted extensive research on this basis. In 2015, Ronneberger et al. introduced U-Net [21], a model that employs a U-shaped encoder-decoder architecture and incorporates skip connections between the encoder and decoder. This design facilitates effective fusion of multi-scale features within the decoder, leading to enhanced segmentation performance. In 2016, Badrinarayanan et al. introduced SegNet [22], a model that follows the structure of an encoder and decoder. Notably, the pooling layer in decoder creates an index and uses the index for upsampling, making the model more efficient.

Subsequent researchers have also made numerous improvements to CNN in terms of enhancing the field of reception and combining multiple scales information. For instance, Chen et al. introduced the Deeplabv3+ [23], which uses dilated convolutions with different rates to build the Atrous Spatial Pyramid Pooling (ASPP), alleviating contradiction between image resolution and limited receptive fields. Zhao et al. proposed PSPNet [24], which uses adaptive pooling operations at different scales to construct the Pyramid Pooling Module (PPM), effectively merging information from various magnification levels and sub-regions. Zhou et al. proposed UNet++ [25], which combined features from different levels and used a flexible network structure with deep supervision, significantly reducing the number of parameters while maintaining acceptable accuracy. However, UNet++ fuses information only with the next layer, leaving the previous layer’s information unutilized. Consequently, the Decoder section still lacks finer granularity. Huang et al. proposed UNet3+ [26], which directly combines feature maps of different scales using full-size skip connections, better preserving and reconstructing the original features of the image, but full-scale skip connections require a significant amount of computational resources.

**Fig 1:**
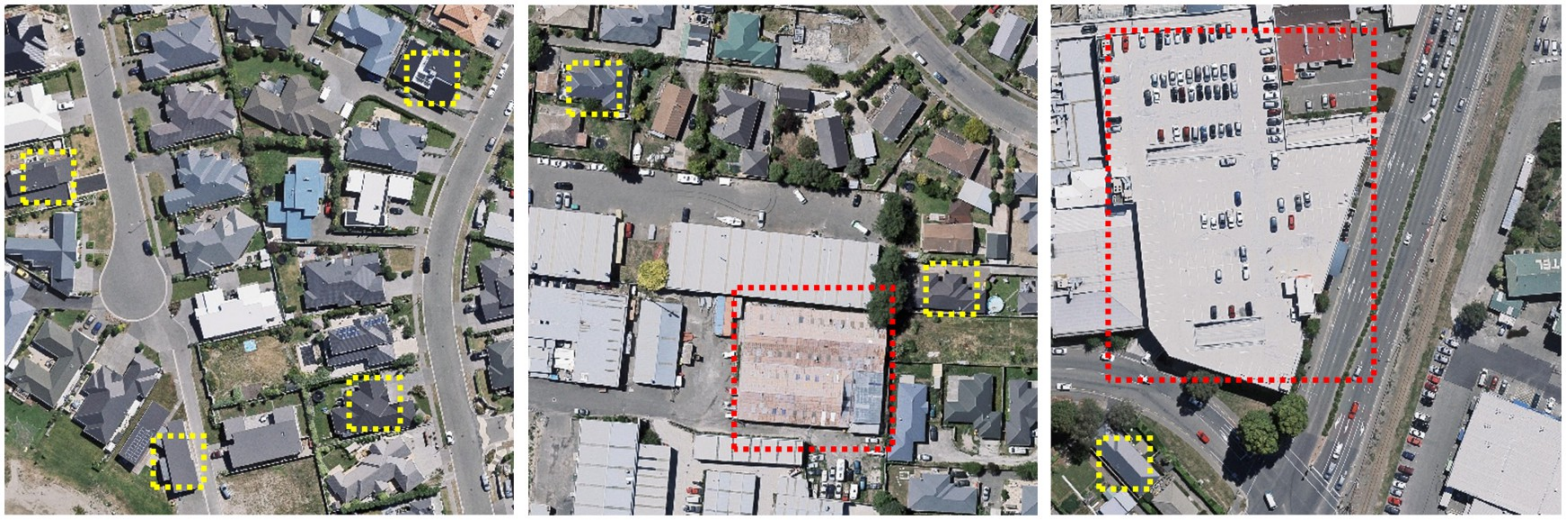
Long range and short range context information perception graph. The yellow area represents the short range contextual information perceived by CNN, while the red area represents the long range contextual information that convolution cannot perceive.(Republished from Ji S et al. under a CC BY license, with permission from Ji S, original copyright 2018.).

Although the aforementioned CNN-based techniques have demonstrated significant advancements in building extraction, the majority of enhancements are centered around the fundamental convolution operation, such as the dilated convolution [27] and deformable convolution [28] methods, which have indeed enhanced the network’s capacity to capture global information to some extent but are still constrained by the localized nature of convolution operations. These methods perform reasonably well in extracting small buildings, but their ability to extract medium to large buildings is severely limited, as shown in Fig 1. Medium to large buildings possess more intricate structures and larger scales, often resulting in unsatisfactory results when these extraction methods are applied. Furthermore, Wang et al. introduced a novel approach by integrating a CNN based detection module with a support vector machine (SVM) based classification module, extracting and fusing multi-scale features from densely distributed buildings in SRS images [29].

To capture global contextual information from images, researchers have proposed introducing attention mechanisms into convolutional neural networks, such as channel attention, and non-local attention. Hu et al. introduced SENet [30], which incorporates a Squeeze-and-Excitation block to calculate channel weights and dynamically, allowing the network to focus on key characteristics of feature maps. Building on SENet,convolutional block attention module (CBAM) [31] introduces global pooling to obtain spatial and channel global information and combined channel attention and spatial attention, effectively enhancing crucial features while suppressing irrelevant ones. Fu et al. proposed DANet [32], which employs a distinct self-attention method compared to CBAM, combining spatial attention blocks and channel attention blocks in parallel captures global message within FCN. Furthermore, recent remote sensing image segmentation tasks have seen the emergence of excellent attention mechanism designs. For instance, Liu et al.’s MSNet [33] introduced a novel MAM (Multi-Attention Mechanism) that effectively constrains spatial and channel dependencies. Raza et al’s EUNet [34] final fusion strategy based on spatial and channel attention serves as a relay to refine multi-scale features and avoid subsampling losses. Zhou et al.’s BOMSC-Net [35] employed a special skip connection to reweight different regions of low-level features in the encoding phase, achieving weight redistribution through attention gate (AG) to make the network focus on areas of interest while suppressing irrelevant background.

Some researchers have introduced the Transformer structure, which is not limited by local operations and excels at modeling global contextual information. Transformer has demonstrated impressive results across a range of natural language processing applications, such as BERT [36] and GPT [37]. Dosovitskiy et al. first introduced the Transformer structure into computer vision tasks and proposed the ViT [38]. Unlike convolutional neural networks, ViT is capable of converting 2D images into 1D sequences and has strong global contextual information modeling capabilities. However, due to its large computation cost and low output resolution, there are limitations in practical applications. To overcome these obstacles, researchers have optimized the Transformer design in the context of ViT. The Swin Transformer [39], developed by Liu et al., combines sliding window operations with a layered architecture, effectively reducing computing complexity.

With the goal of improving the performance of image segmentation, scholars have explored the integration of Transformer architectures into segmentation networks, and there have been two main research branches of Transformer-based segmentation networks. One approach is the pure Transformer structure, such as SwinUNet [40] proposed by Cao et al which uses the Swin Transformer as the primary feature extraction module and adopts an encoding and decoding structure similar to Unet. This integration improves the computational efficiency and global context modeling ability of the network, but lags behind traditional CNN in terms of proficiency in extracting local feature information. The other research branch is the hybrid structure that combines Transformer with CNN, such as TransUNet [41] proposed by Chen et al which adopts the overall structure of Unet and incorporates the Transformer into the innermost depths of network enables extraction of global contextual information from CNN, while in the earlier stages of the network, information extraction is insufficient for images containing rich low-level semantic features. Furthermore, in remote sensing image segmentation tasks, models such as Yuan et al.’s MSST-Net [42], Wang et al.’s DC Swin [43], and Wang et al.’s SwinUperNet [44] all employ SwinTransformer in the encoding phase and hierarchical CNN decoding in the decoding phase. However, they do not effectively leverage CNN’s local feature extraction capabilities during the encoding phase. Cui et al. integrated a convolutional attention module into the original Swin Transformer as the backbone of the encoder, in the decoder stage, adopted a multilevel segmentation structure based on CNN, enhancing the capability to extract both global and local features to a certain extent [45].

To address the above-mentioned issues, this article proposed a new type of Transformer-based image segmentation hybrid model (STransU2Net), which is able to capturing global contextual message while preserving fine-grained local details. For Transformer branch, we utilized the efficient global modeling ability of the Swin Transformer and incorporated it simultaneously into the deep and shallow layers of network to obtain full global contextual message. For CNN branch, we constructed a novel downsampling module and spatial channel attention mechanism in U2Net [46] structure to extract detailed features of the feature map. With these improvements, the integration capability of our model’s local messages from CNN and global messages from Transformer has been enhanced. The following notable contributions are highlighted in this article:

1. We present a unique Bottleneck Pooling Block (BPB) that replaces the typical pooling layer during the downsampling step with a dual-branch convolution layer and pooling layer to preserve edge information lost by the picture through the pooling layer.
2. We propose a novel CNN-based spatial and channel attention mechanism (CSAB), which is used throughout both the encode and decode steps to enhance information of the target location and suppress irrelevant information.
3. We simultaneously incorporate Swin Transformer Blocks into the skip connections with both deep and shallow levels of CNN, thus the feature maps from the CNN encoder can be passed into the Swin Transformer Block to obtain global contextual information.

## Materials and methods

### STransU2Net structure

**Fig 2:**
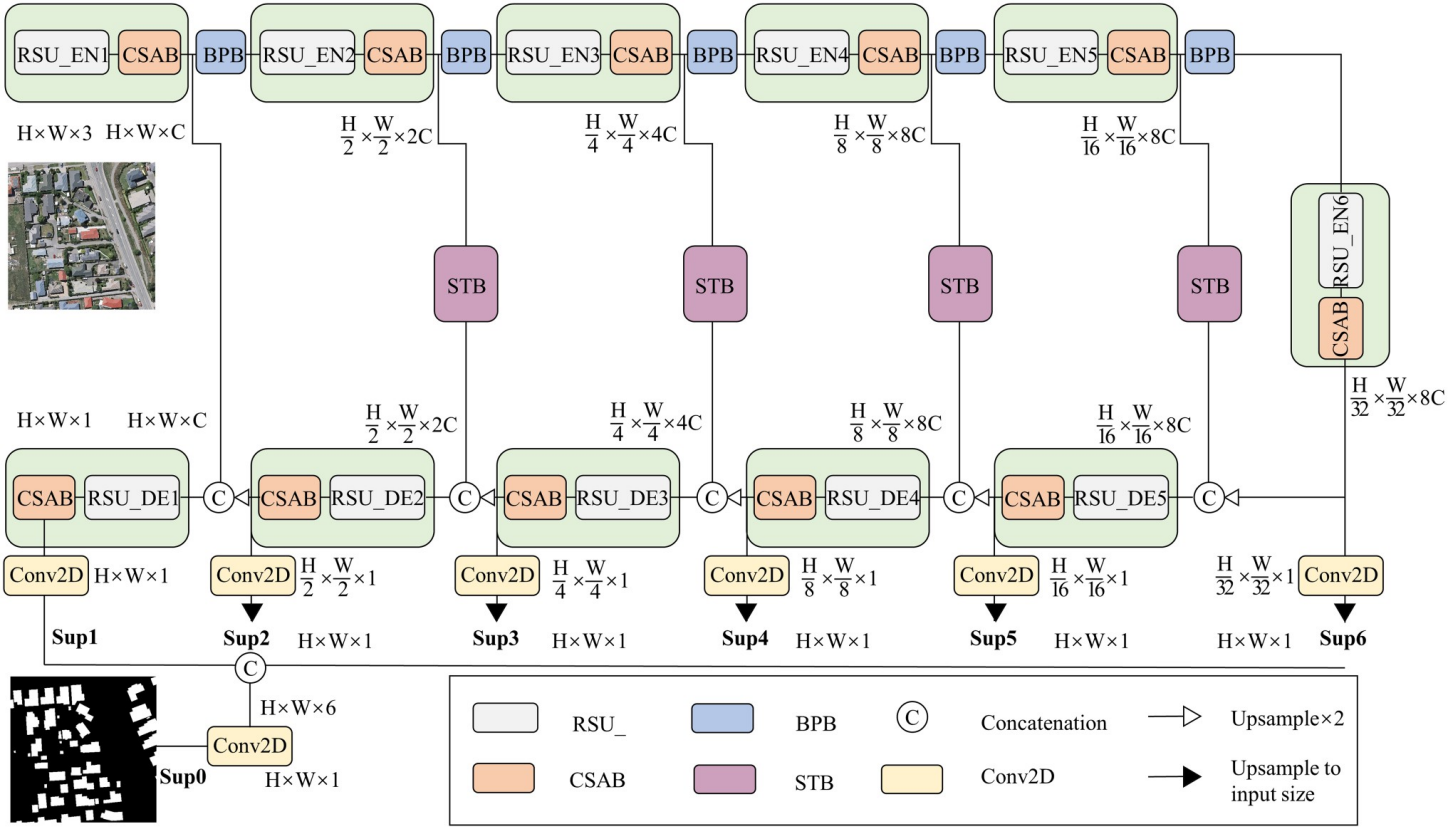
STransU2Net structure diagram. (Republished from Ji S et al. under a CC BY license, with permission from Ji S, original copyright 2018.).

**Fig 3:**
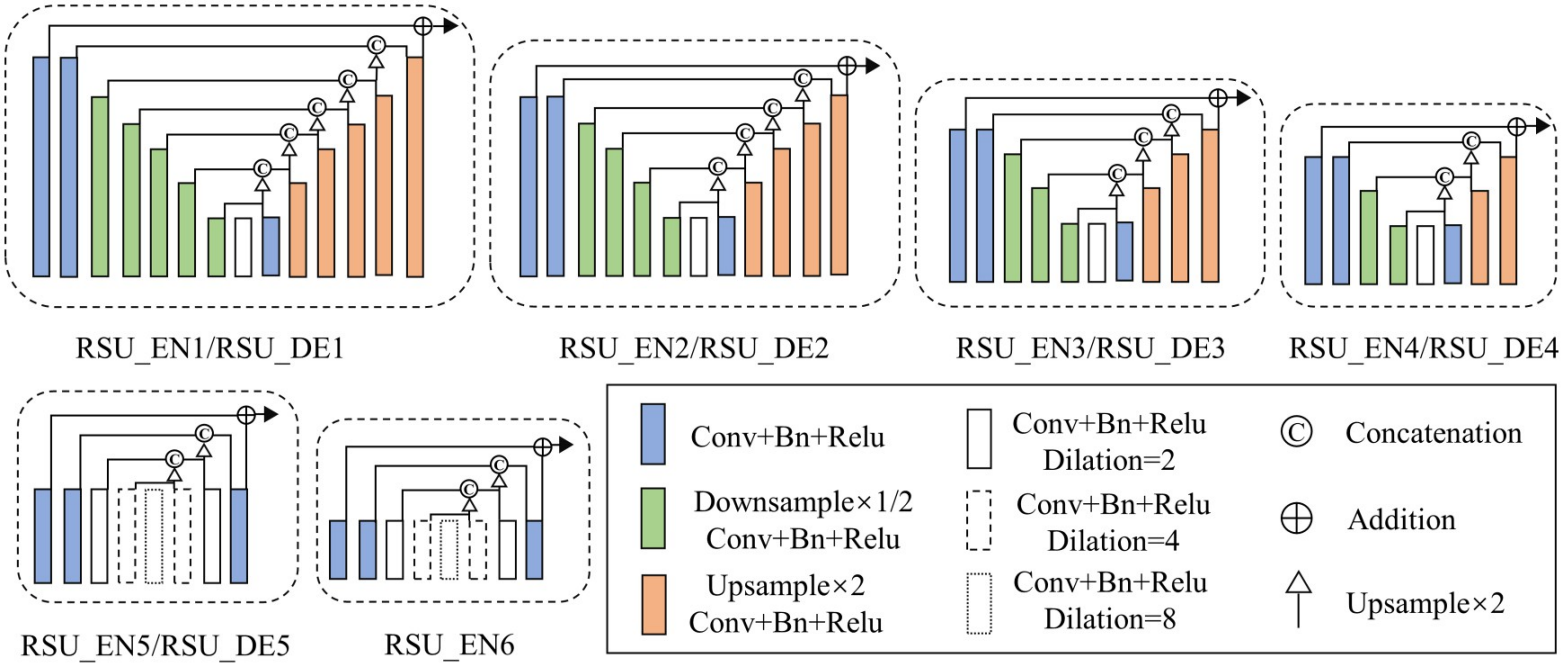
RSU structure diagram.

The STransU2Net proposed in this paper is a Transformer-based image segmentation hybrid model. Illustrated in Fig 2, 11 U-shaped residual blocks (RSUs) [46, 47] make up the encoder and decoder’s main body, with a CSAB attention mechanism added after each RSU to provide more attention to important parts of the image and enhance the learning of building features, the RSU structure is illustrated in Fig 3. In the encoding stage, down-sampling is performed through BPB to compensate for the large amount of details lost during maxpooling layer.

Additionally, Swin Transformer Block (STB) is installed at the skip-connection positions, which comprise two multi-head self-attention modules. The previous module applies window multi-head self-attention mechanism (W-MSA), whereas the latter module employs shifted-window multi-head self-attention mechanism (SW-MSA), STB divides the image into multiple local blocks and introduces a sliding window between these blocks, facilitating communication among them. This innovative approach allows for the capture of long-range dependencies across these blocks. As the network deepens, the number of local blocks within the window continuously increases, significantly improving the ability to extract global contextual information.

The fusion process between CNN and Transformer in STransU2Net can be summarized in the following manner:the feature maps are first undergo the RSU and then divided into the CNN and Transformer paths. For the CNN path, the feature maps are progressively encoded through later RSUs and downsampling operations. For the Transformer path, in the network’s first layer,in order to better preserve their low-level features, the feature maps are not passed through STB. However, feature maps are routed through STB starting with the second skip connections layers. Then, the information is propagated to the CNN decoder.

During the deep supervision step, Six feature maps are produced by the RSU as outputs, Using 3x3 convolutions and sigmoid activation, six segmentation images (Sup1, Sup2, Sup3, Sup4, Sup5, Sup6) are generated. Sup2 to Sup6 are upsampled to match the original image size. These six images are then concatenated and processed with a 1x1 convolution to generate the final segmentation image, Sup0. Finally, Sup0 along with Sup1 to Sup6, is used for network supervision during training.

### Bottleneck Pooling Block(BPB)

**Fig 4:**
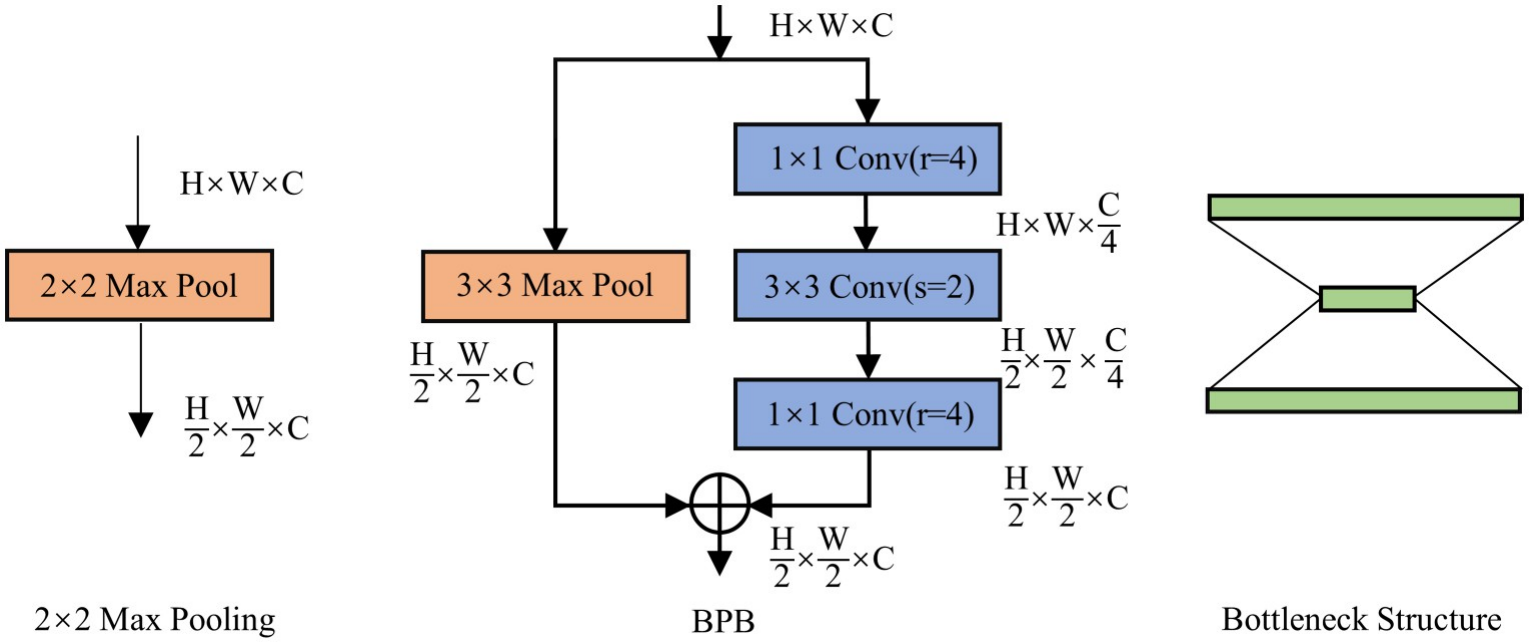
Bottleneck Pooling Block structure diagram.

In the encoding stage of a general CNN, the feature map needs to be downsized from H to H/2 by a maxpooling layer of size 2×2 with a stride of 2. However, this downsampling process results in a significant loss of edge information as the feature map passes through the max-pooling branch. To overcome this issue, this research proposes Bottleneck Pooling Block (BPB) by adding a convolutional branch next to the maxpooling branch to compensate for the lost information during downsampling, as illustrated in Fig 4.

By incorporating a convolutional branch into the BPB, the model can retain the downsampling effect of pooling operations while giving heightened consideration to edge information. This mitigation strategy effectively prevents the blurring and erosion of edge features. The convolutional branch learns appropriate parameters to retain essential details, thereby enhancing the model’s sensitivity to edges, textures, and other critical information. This improvement significantly enhances the performance of bottleneck pool blocks in building extraction tasks.

In particular, with a stride of 2, a convolutional branch of size 3×3 is added next to the maxpooling branch. Additionally, the original 2×2 maxpooling is replaced with a 3×3 maxpooling obtain increase the perception area. Considering that adding a convolutional branch increases computational complexity, the paper designs a bottleneck structure, which compresses the channel of the image before convolution with a compression ratio of r. This is done by adding two 1×1 convolutions with stride 1, which reduces the channel number to C/4 before the feature map goes through the 3×3 convolution, assuming that the original feature map has a channel number of C. After the 3×3 convolution, the channel number is increased back to C through another 1×1 convolution. Finally, an addition operation integrates the feature map from the Maxpooling branch with the one derived from the convolutional branch. The output of this fused feature map is then provided to the subsequent layer of the RSU.

### Channel And Spatial Attention Block(CSAB)

This paper proposes an efficient attention mechanism module based on channel and spatial dimensions of feature maps, called Channel And Spatial Attention Block (CSAB). The channel attention primarily focuses on the correlation between different channels within the feature map, where some channels may contain more useful information while others may contain noise or irrelevant data. And, spatial attention addresses the correlation between different positions within the feature map, with a particular emphasis on the importance of information within building regions. The goal of both forms of attention is to enhance the network’s performance adaptively, either by assigning different weights to channel features or by highlighting significant spatial locations within the feature map.

**Fig 5:**
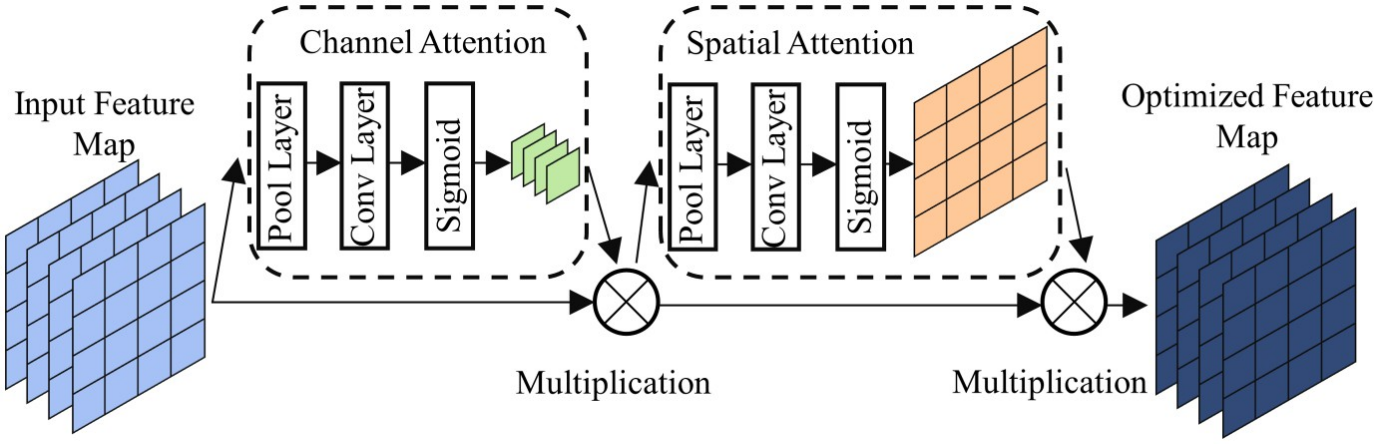
Structure of CSAB.

This block has a compact parameter size and significantly enhances the network’s segmentation capability without significantly increasing computational complexity. The design of CSAB is illustrated in Fig 5. Initially, the feature map produced by RSU undergoes processing in the channel attention block. This module produces maps of channel message, which are combined with the feature map. Subsequently, it undergoes the Spatial Attention module, generating spatial weight map is combined with the weighted feature map through the channel attention block. Finally, the results are obtained by combining the two attention mechanisms.

**Fig 6:**
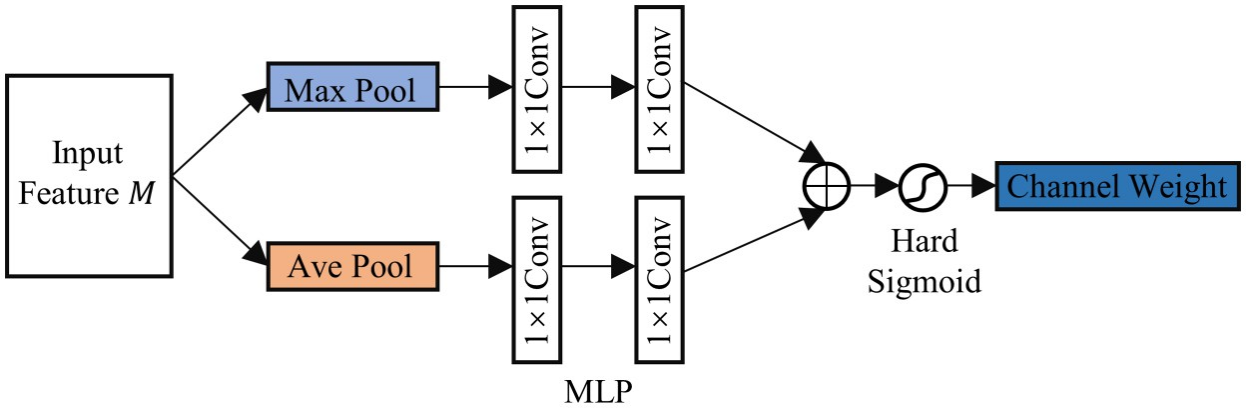
Structure of channel attention block.

The input feature map is used by the channel attention block, as illustrated in Fig 6. The feature map first goes via global maximum and global average pooling, and then through the shared fully connected layer (MLP) compressing the number of channels to C/r and then back to C, Where r is the proportion of compression and C is the original amount of channels. the features obtained from the MLP are added together and activated using the Hard Sigmoid activation function, resulting in the generation of the channel attention weight map. The formula can be expressed as as presented below:

$$\begin{array}{c} {CSAB}_{c}(M)=\sigma \left(MLP\left(M_{avg}^{c}\right)+MLP\left(M_{max}^{c}\right)\right) \end{array}$$ (1)

In the equation, M stands for the Input Feature Map, *α* stands for the Hard Sigmoid activation function, and $M_{avg}^{c}$ and $M_{max}^{c}$ representing global average pooling and global maximum pooling on feature map channels, respectively.

**Fig 7:**
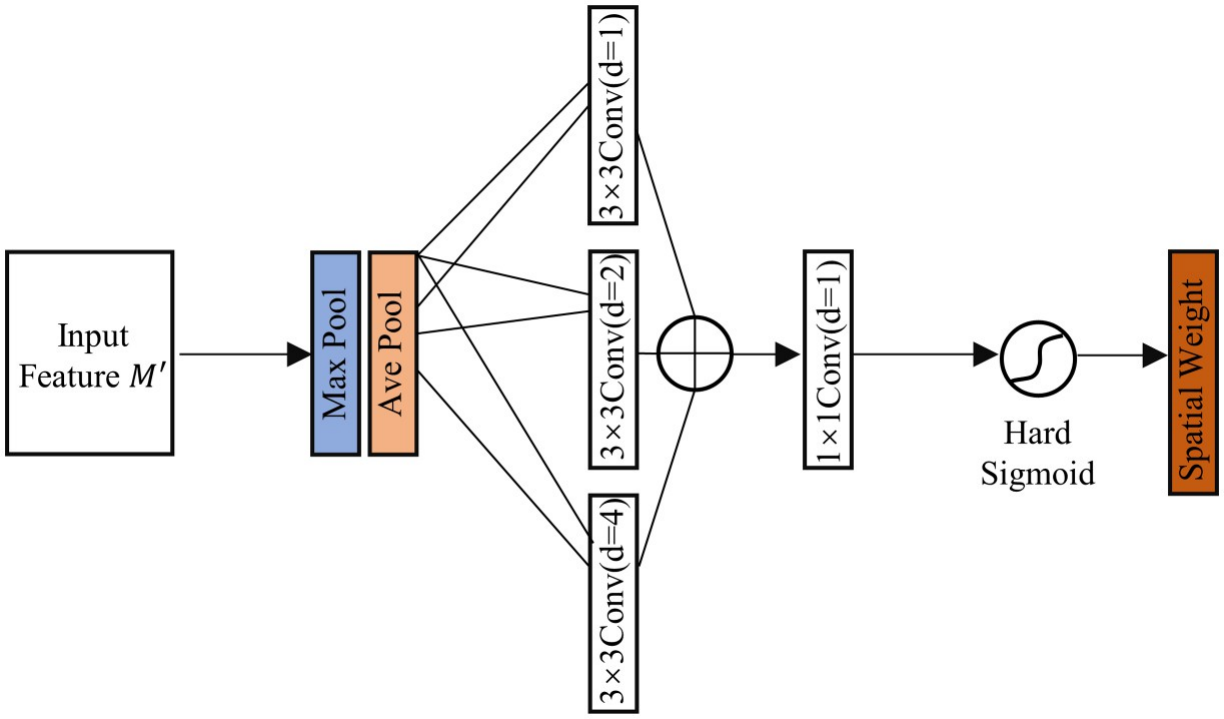
Structure of Spatial Attention Block.

The structure of the Spatial Attention Block is illustrated in Fig 7. First, the feature maps pass through global max pooling and global average pooling based on channels for each pixel. Then, they are further processed individually using 3×3 grouped convolutions with dilation rates of 1, 2, and 4, and the results are summed to aggregate them. Subsequently, feature fusion is performed with a 1×1 convolutional layer whose output dimension is 1. The Hard Sigmoid activation function is then applied to the results to create the spatial attention weight map. The formula can be expressed as as presented below:

$$\begin{array}{c} {CSAB}_{s}(M)=\sigma \left(n_{1}^{3\times 3}\left({(n}_{1}^{3\times 3}+n_{2}^{3\times 3}+n_{4}^{3\times 3}\right)\left(M_{avg}^{s}+M_{max}^{s}\right)\right)) \end{array}$$ (2)

In the equation, $n_{1}^{3\times 3}$ denotes the 3×3 convolution with a dilation factor of 1, and $M_{avg}^{s}$ and $M_{max}^{s}$ representing global average pooling and global maximum pooling on feature map spaces, respectively.

### Experimental details

#### Experimental environment

In this paper, there were 100 epochs in the training process. We utilized the Poly learning rate decay method with a decay rate of 0.9, a batch size of 4, and an initial learning rate of 0.01, decayed to 0. SGD was the model optimizer employed, with weight decay set to 4 × 10^−5^ and momentum set to 0.9.

#### Datasets

Two publicly accessible building extraction datasets from the WHU building dataset, namely the Aerial imagery dataset and the Satellite dataset II(East Asia), were used in the experiments of this study to evaluate the effectiveness of STransU2Net.

1. Aerial imagery dataset [48]: The selected images in this dataset are located in Christchurch, New Zealand. The entire area is divided into 8,189 images of size 512x512 pixels. The dataset utilized in this work included 4,736 training images, 1,036 validation images, and 2,416 testing images altogether.
2. Satellite II dataset(East Asia) [48]: The dataset comprises six adjacent satellite images covering 860 square kilometers in East Asia. The entire image is seamlessly cropped into 512x512 pixels. In order to enhance the efficiency of the experiment, We filtered out images containing building information and divided the dataset into 2,508 training images, 627 validation images, and 903 testing images.

#### Data enhancement

To improve image quality, enhance image features, and increase model accuracy, this paper used the following data augmentation and enhancement techniques on the selected two datasets: random horizontal flip, random vertical flip, random rotation of 15 degrees, random resizing, Gaussian blur and sharpening.

#### Loss function

This paper selects Binary Cross Entropy (BCE) as the loss function of the network, and the formula for cross-entropy is as follows:

$$\begin{array}{c} l_{n}=-\left(y_{n}\;\text{log}\;z_{n}+(1-y_{n})\;\text{log}\;(1-z_{n})\right) \end{array}$$ (3)

$$\begin{array}{c} loss(z,y)=mean\left(l_{0}\cdots l_{N-1}\right) \end{array}$$ (4)

where N represents the number of pixels, *z*_*n*_ represents the probability of predicting the nth pixel as a positive example, that is the probability of predicting the pixel as a building, and *y*_*n*_ represents the label of the nth pixel.

#### Evaluation indicators

To better measure the algorithm’s performance, the following commonly used evaluation metrics are selected for the satellite image building segmentation work [3–5]: IoU, F1, Precision, and Recall.

## Results and discussion

### Ablation experiment

We initially conducted experiments to evaluate the upsampling method. Subsequently, in order to demonstrate the efficiency of Swin Transformer Block(STB), Bottleneck Pooling Block(BPB), and Channel And Spatial Attention Block(CSAB), a series of validation experiments were conducted on the Aerial imagery dataset.

#### Upsampling Block

To evaluate the impact of different upsampling methods on the experimental results, a series of experiments were conducted, and the results are presented in Table 1. In the table, BI represents the use of bilinear interpolation exclusively, while TC signifies the exclusive use of transposed convolution. BI-TC denotes the simultaneous use of both bilinear interpolation and transposed convolution.

**Table 1:** Aerial imagery dataset: Ablation experiment results of Upsampling Block.

Method	IoU(%)	F1(%)	Params(MB)	Flops(G)
BI	**90.45**	**94.93**	**168.05**	**150.69**
TC	90.00	94.57	177.37	168.98
BI-TC	90.23	94.78	177.37	168.98

Through experiments, we found that the TC yielded IoU and F1 scores of 90.00% and 94.57%, respectively, indicating the poorest performance. However, when we combined the bilinear interpolation branch on top of TC and added the feature maps from both branches, the BI-TC showed improvements of 0.23% in IoU and 0.21% in F1. Additionally, the BI, which involves fewer floating-point operations and parameters, exhibited an increase of 0.45% in IoU and 0.36% in F1. This suggests that introducing transposed convolution in the upsampling stage can negatively impact segmentation accuracy. Consequently, subsequent experiments were conducted using the BI.

#### Swin Transformer Block(STB)

Experiments on the downsampling step of BPB were conducted in order to evaluate the effect of BPB on the experimental results, Table 2 presents the findings of experiments. The STB parameters were set with a window size of 8 and a number of heads of 8. Table 1 presents the findings of the experiment, with NoSTB represents no STB, STB×2 represents repeating STB twice, and STB×1 represents repeating STB once.

**Table 2:** Aerial imagery dataset: Ablation experiment results of STB.

Method	IoU(%)	F1(%)	Precision(%)	Recall(%)
NoSTB	90.45	94.93	95.44	94.43
STB×2	90.53	94.99	95.50	94.49
STB×1	**90.61**	**95.07**	**95.61**	**94.54**

When no STB was added, both IoU and F1 obtained poor results. After adding STB, the performance improved, and there was a 0.16% increase in IoU and a 0.14% increase in F1. It can be seen that adding STB, which integrates global information, can effectively enhance the network’s information extraction capability. Furthermore, we repeated the STB twice, but the effect was not as good as repeating once, where there was a 0.08% increase in IoU and a 0.06% increase in F1. This observation suggests that due to the small size of the building dataset and the absence of pre-training parameters, increasing the number of STB repetitions does not significantly improve effectiveness.

#### Bottleneck Pooling Block(BPB)

To verify the effect of BPB on experimental results, we conducted experiments on the BPB in the downsampling stage, and the experimental results are shown in Table 3. BPB2 represents that both the convolutional and pooling operations have a size of 2×2, and BPB3 represents that both the convolutional and pooling operations have a size of 3×3.

**Table 3:** Aerial imagery dataset: Ablation experiment results of BPB.

Method	IoU(%)	F1(%)	Precision(%)	Recall(%)	Params(MB)	Flops(G)
NoBPB	90.45	94.93	95.44	94.43	**168.05**	**150.69**
BPB2	90.52	95.01	95.48	94.55	169.26	152.18
BPB3	**90.68**	**95.12**	**95.54**	**94.71**	169.27	152.19

Through experiments, we found that compared with NoBPB, BPB3 improved a 0.23% in IoU and a 0.19% in F1, while the increase in Params was small, indicating the effectiveness of combining a convolutional branch to supplement information next to the max pooling branch, as well as the economy of designing the convolutional branch as a bottleneck structure. In addition, the effect of BPB2 was not as good as BPB3, BPB2 compared to NoBPB slightly improved a 0.07% in IoU and a 0.08% in F1.

#### Channel And Spatial Attention Block (CSAB)

To investigate the effects of the insertion position and compression ratio of CSAB on the experimental results, we conducted experiments on CSAB, and Table 4 presents the findings of experiments. E represents that CSAB is added to the encoder stage, ED represents that CSAB is added to both the encoder and decoder stages, and 4 and 16 represent the compression ratio.

**Table 4:** Aerial imagery dataset: Ablation experiment results of CSAB.

Method	IoU(%)	F1(%)	Precision(%)	Recall(%)
NoCSAB	90.45	94.93	95.44	94.43
CSAB-E4	90.52	95.02	95.41	94.64
CSAB-ED4	90.63	95.14	95.56	94.72
CSAB-E16	90.57	95.08	95.48	94.69
CSAB-ED16	**90.74**	**95.22**	**95.69**	**94.75**

By observing the experimental findings, it can be concluded that adding CSAB to the network can greatly increase segmentation accuracy. Compared to NoCSAB, CSAB-ED16 improved a 0.29% in IoU and a 0.29 in F1. Adding CSAB to both the encoder and decoder stages is better than adding it to the encoder stage alone. Compared to CSAB-E16, CSAB-ED16 improved a 0.17% in IoU and a 0.14% in F1. Similarly, compared to CSAB-E4, CSAB-ED4 improved a 0.11% in IoU and a 0.12% in F1. Indicating that attention mechanisms are also needed in the network decoding stage to enhance feature learning.

In addition, the compression ratio of 16 has a better effect than the compression ratio of 4. Compared to CSAB-ED4, CSAB-ED16 improved a 0.11% in IoU and a 0.08% in F1. Similarly, compared to CSAB-E4, CSAB-E16 improved a 0.05% in IoU and a 0.06% in F1. Indicating that a low compression ratio can cause the network to overlearn, affecting segmentation performance.

Additionally, to validate the impact of global maximum pooling branche and global average pooling branche within the channel attention block, we conducted experiments on the CSAB-ED16 model, and the results are presented in Table 5. Here, Max represents the global maximum pooling branch, and Ave represents the global average pooling branch.

**Table 5:** Aerial imagery dataset: Ablation experiment results of CSAB-ED16.

Method	IoU(%)	F1(%)	Precision(%)	Recall(%)
CSAB-Max	90.53	94.99	**95.76**	94.24
CSAB-Ave	90.62	95.08	95.63	94.54
CSAB-Max-Ave	**90.74**	**95.22**	95.69	**94.75**

Upon examining the experimental results, it becomes evident that removing either the Max branch or the Ave branch from CSAB-Max-Ave has an influence on the segmentation performance. Specifically, when the Max branch is removed, CSAB-Ave exhibits a reduction of 0.12% in IOU and 0.14% in F1 score compared to CSAB-Max-Ave. Similarly, when the Ave branch is omitted, CSAB-Max experiences a decline of 0.21% in IOU and 0.23% in F1 score compared to CSAB-Max-Ave. This indicates that introducing the Max and Ave branch within the channel attention block in CSAB-ED16 enables a more comprehensive extraction of channel information from the feature maps.

#### STransU2Net

According to the separate experimental results of the three blocks, as shown in Table 6, we selected the method with the maximum improvement from each block for the next experiment: STB×1 with one repetition, BPB3 with a convolution and pooling size of 3×3, and CSAB-ED16 with a compression ratio of 16 added in both the encoding and decoding stages.

**Table 6:** Aerial imagery dataset: Ablation experiment results of STransU2Net.

BPB3	STB×1	CSAB-ED16	IoU(%)	F1(%)	Precision(%)	Recall(%)
-	-	-	90.45	94.93	95.44	94.43
-	✓	-	90.61	95.07	95.61	94.54
✓	-	-	90.68	95.12	95.54	94.71
-	-	✓	90.74	95.20	95.89	94.52
✓	-	✓	90.79	95.16	95.78	94.55
-	✓	✓	90.91	95.21	95.65	**94.78**
✓	✓	-	90.86	95.18	95.68	94.68
✓	✓	✓	**91.04**	**95.31**	**96.19**	94.45

After comparing the experimental results of the combination of the three modules, the fusion of BPB and CSAB improved a 0.34% in IoU and a 0.23% in F1, the fusion of BPB and STB improved a 0.46% in IoU and a 0.28% in F1, the fusion of CSAB and STB improved a 0.41% in IoU and a 0.25% in F1. The results indicate that incorporating the STB for obtaining global information and CSAB for enhancing target position information can effectively improve segmentation accuracy. Based on this, BPB is added to preserve for the message loss in downsampling stage, leading to the proposed STransU2Net network that integrates BPB, STB, and CSAB.

### Training experiments

**Fig 8:**
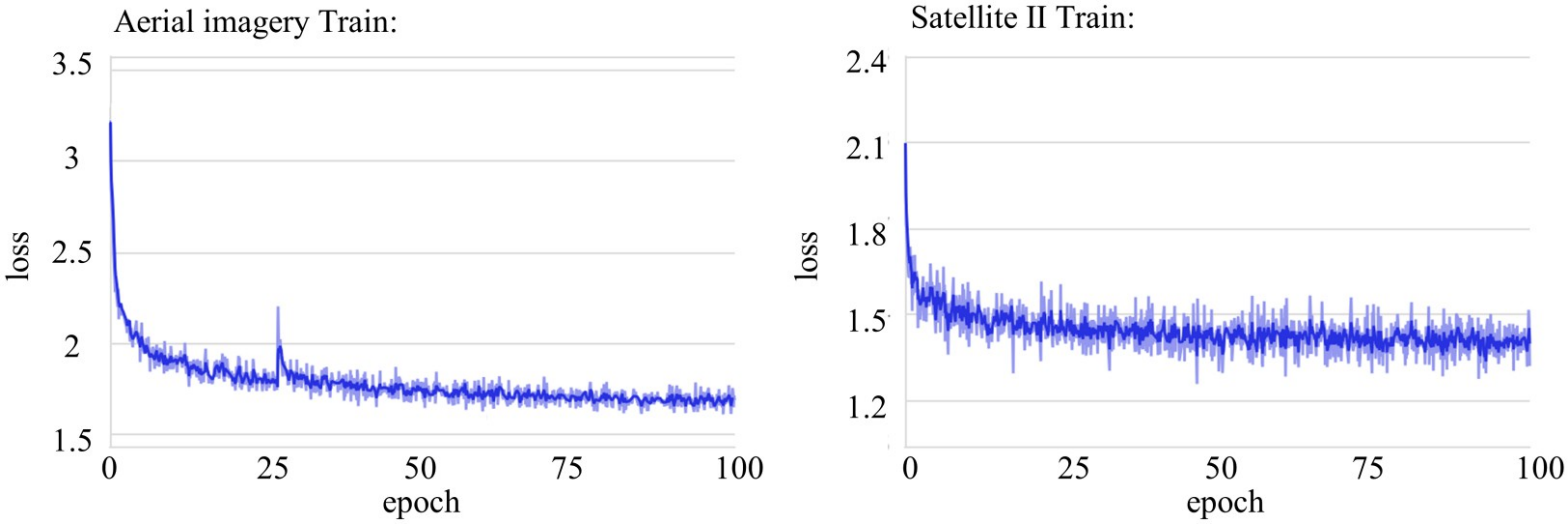
Aerial imagery dataset and Satellite II dataset: Training loss curve.

Fig 8 presents the two loss curves for model training on the Aerial imagery dataset and the Satellite II dataset, respectively. The horizontal axis represents the number of training epochs, while the vertical axis represents the loss values. From the loss curves, it can be observed that as the number of epochs increases, the training loss gradually decreases, indicating that the model is gradually converging on the training dataset. When the training reaches approximately 70 iterations, the loss function converges to its minimum value. However, to ensure avoidance of local minima, we continued training for an additional 30 rounds, bringing the total number of training epochs to 100, thus confirming the global minimum.

### Comparative experiment

Both the Aerial imagery dataset and the Satellite II dataset were used in our experiments to demonstrate the advantages of STransU2Net in building segmentation work, and compared it with efficient CNN segmentation algorithms and recently proposed Transformer segmentation algorithms. Table 7 presents the findings of experiments. On the Aerial imagery dataset, STransU2Net achieved evaluation metrics IoU and F1 of 91.04% and 95.31%, respectively. On the Satellite II dataset, STransU2Net achieved evaluation metrics IoU and F1 of 59.09% and 74.24%, respectively.

**Table 7:** Aerial imagery dataset and Satellite II dataset: Comparative experiments results between STransU2Net and different models.

Method	Aerial imagery		Satellite II	
	IoU(%)	F1(%)	IoU(%)	F1(%)
Unet [21]	87.53	93.06	52.46	68.82
PSPNet [24]	87.78	93.15	56.31	72.05
DeepLabv3+ [23]	88.11	93.68	48.44	65.27
UNet++ [25]	88.52	93.91	57.77	73.23
AttenUNet [49]	89.60	94.51	57.65	73.13
DANet [32]	88.98	94.06	55.47	71.36
UNet3+ [26]	89.29	94.34	57.60	73.10
U2Net [46]	90.45	94.93	58.20	73.63
EUNet [34]	88.32	93.76	57.27	72.64
MSNet [33]	89.07	93.96	58.34	73.69
BOMSC-Net [35]	90.15	94.80	58.58	73.82
TransUNet [41]	88.17	93.71	54.34	70.42
SegFormer [50]	86.62	92.83	51.22	67.75
SwinUNet [40]	88.76	94.04	52.57	68.91
MSST-Net [42]	88.00	93.68	56.86	72.57
DC-Swin [43]	89.86	94.49	58.63	73.92
Swin-UperNet [44]	89.64	94.40	58.82	74.08
STransU2net	**91.04**	**95.31**	**59.09**	**74.24**

Compared to CNN-based networks such as UNet [21], PSPNet [24], Deeplabv3+ [23], UNet++ [25], UNet3+ [26], U2Net [46], AttentionUNet [49], DANet [32], EUNet [34], BOMSC-Net [35] and MSNet [33], the proposed STransU2Net’s findings demonstrate its greater effectiveness. Although the pyramid pooling block(PSP) of PSPNet,the atrous spatial pyramid pooling block(ASPP) of Deeplabv3+,BOMSC-Net’s multi-scale context-aware module (MSCAM) and MSNet’s multiregion scale-aware network can extract multi-scale feature maps, their ability to extract global information is not as effective as the Transformer due to the local operation of convolution and pooling. Although UNet++ and UNet3+ use densely connected network structures and multi-scale information utilization, their global information modeling capability is still inferior to the Transformer. EUNet, while incorporating the MBConv module in both the encoding and decoding stages, enhances architectural efficiency but has an impact on segmentation accuracy. Although AttentionUNet and DANet use non-local attention mechanisms to establish long-range dependency relationships, their CNN components lack comprehensive design, resulting in weaker local feature extraction abilities compared to STransU2Net.

Compared with Transformer-based networks SegFormer [50], SwinUNet [40], TransUNet [41], MSST-Net [42], DC-Swin [43] and SwinUperNet [44], the proposed STransU2Net in this research performs superior in terms of experimental findings. Although TransUNet introduces the Transformer Block to obtain global information in the network, it only introduces it in the deep layers and not in the shallow layers. In contrast, the proposed STransU2Net introduces Swin Transformer Block in both deep and shallow layers, which fully utilizes global information. Additionally, Swin Transformer uses sliding windows for self-attention, which results in fewer params and flops compared to Transformer, and STransU2Net’s params are only 54.95% of TransUNet’s. The MSST-Net, DC-Swin, and SwinUperNet models all utilize the SwinTransformer in the encoding stage and adopt a layer-wise CNN decoding in the decoding stage. However, the fusion between CNN and Transformer components is not achieved optimally. SegFormer and SwinUNet are pure Transformer models which demand an enormous quantity of data to train. Since the building dataset for building segmentation images is generally a small to medium-sized dataset and lacks pre-training parameters, SegFormer and SwinUNet did not achieve good experimental results. The proposed STransU2Net in this research is a hybrid model, which does not require pre-training parameters or an enormous quantity of training data to produce decent outcomes.

**Fig 9:**
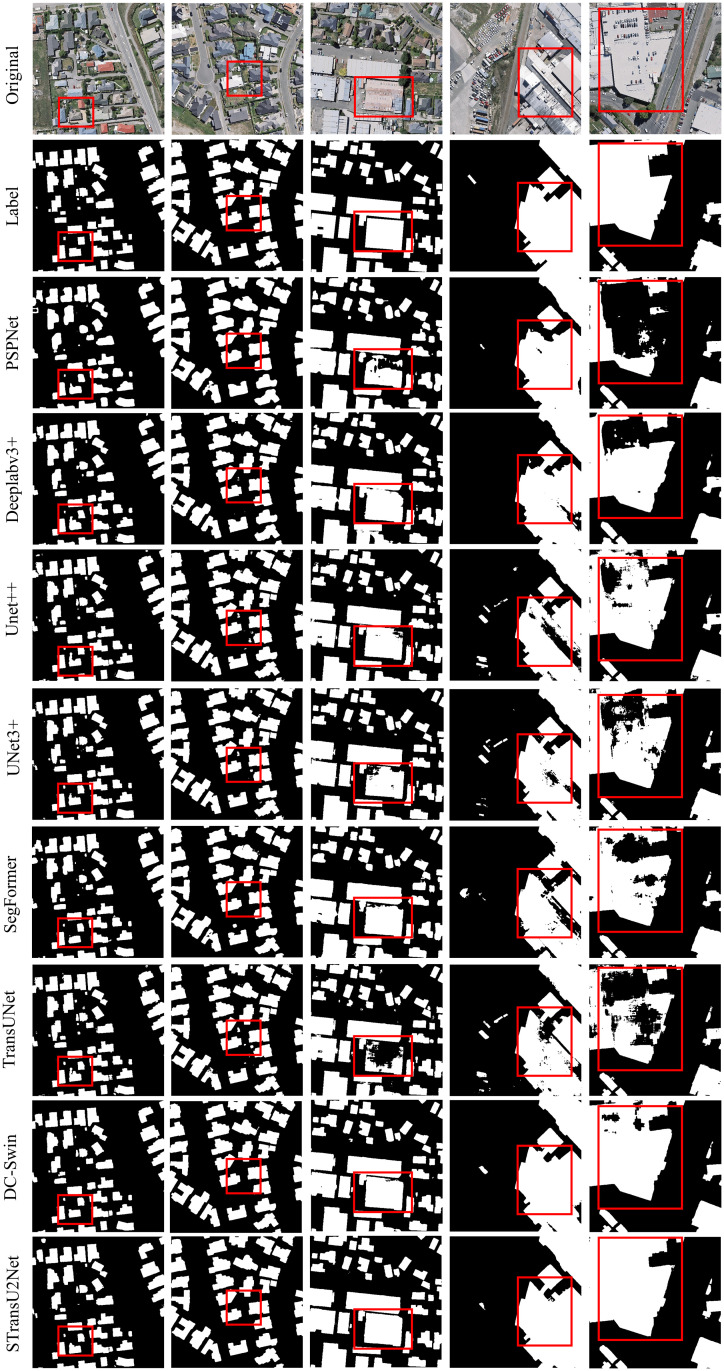
Aerial imagery dataset: Numerous models’ segmentation results(Republished from Ji S et al. under a CC BY license, with permission from Ji S, original copyright 2018.).

**Fig 10:**
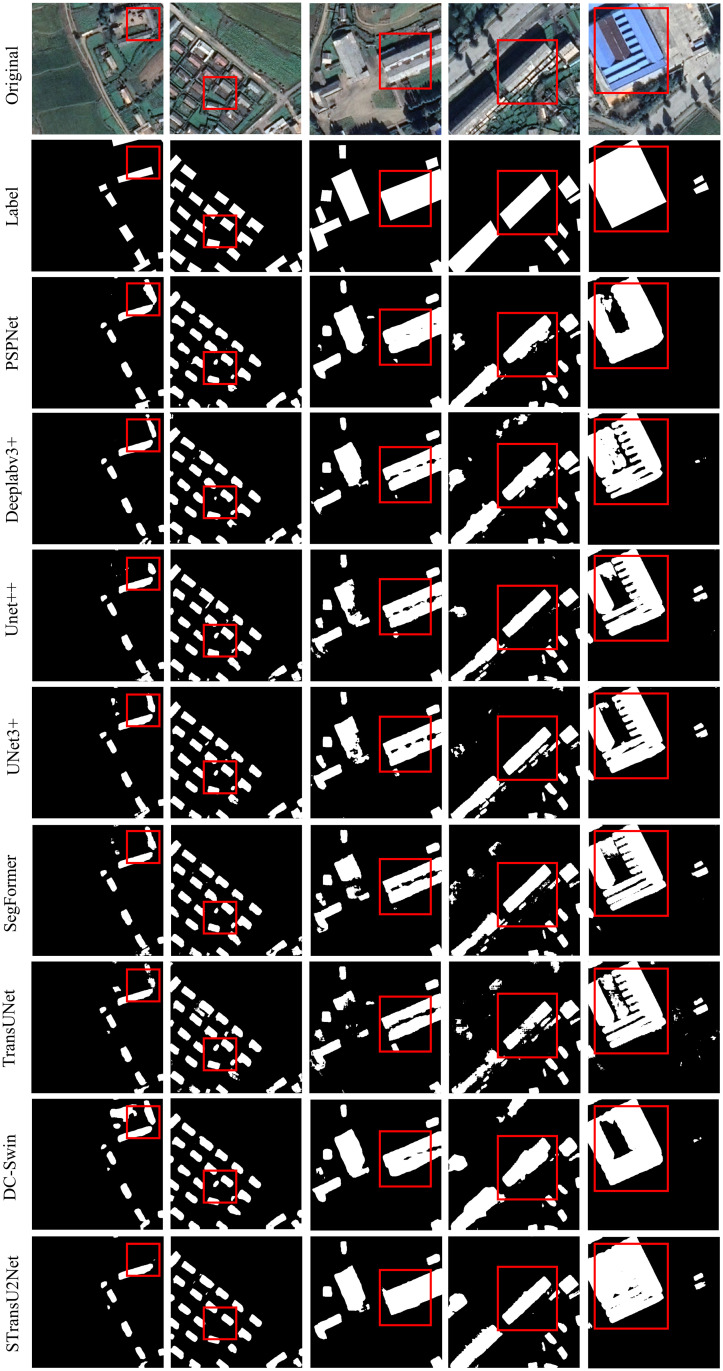
Satellite II dataset: Numerous models’ segmentation results(Republished from Ji S et al. under a CC BY license, with permission from Ji S, original copyright 2018.).

Finally, Figs 9 and 10 show the segmentation results and label comparisons of multiple models on the Aerial imagery dataset and the Satellite II dataset, respectively. Observing the findings of experiments, for small buildings (first and second columns), STransU2Net can effectively preserve the detail information with almost no misclassification or omission. For medium-sized buildings (third and fourth columns), other methods have some holes, while STransU2Net has a more detailed segmentation result, with no holes and more complete boundaries. For large buildings (fifth column), other CNN based and Transformer based methods have large-scale missegmentation, while STransU2Net produces complete results with no large-scale missegmentation. The experimental findings demonstrate that the spatial-channel attention mechanism and downsampling optimization in the CNN network can enhance the ability to extract building edges. Moreover, introducing Transformer with global modeling capabilities can effectively improve the segmentation ability for medium and large buildings.

## Conclusions

This paper proposes a Transformer based hybrid model for building segmentation in satellite images (STransU2Net). We have designed CSAB and BPB in the model to improve the network’s capacity to extract multi-level details and to avoid segmentation errors caused by localized perception. Additionally, we have added Swin Transformer structure in the skip-connection part to increase the network’s capability to extract global message, which successfully extracts buildings of different sizes and preserves their detailed contours, achieving greater robustness and versatility. In future research, we will further explore other ways to combine CNN and Transformer.
